# Ectopic Breast Tissue in the Inguinal Region: A Case Report and Suggestion of Indications for Excision

**DOI:** 10.3389/fsurg.2022.894416

**Published:** 2022-07-05

**Authors:** Jangyoun Choi, Young Bin Yang, Deuk Young Oh

**Affiliations:** Department of Plastic and Reconstructive Surgery, Seoul St. Mary's Hospital, College of Medicine, The Catholic University of Korea, Seoul, Korea

**Keywords:** ectopic, breast, inguinal, neoplasm, hormone-sensitive

## Abstract

We report a rare case of ectopic breast tissue situated in a unique location. A 50-year-old female patient came to our institution complaining of a bulge in the inguinal area. CT was unremarkable other than a benign-looking conglomeration of lymph nodes around the inguinal canal. However, excisional biopsy proved otherwise, with strong expression of breast-related immunohistochemical markers on pathology. Based on histological findings, the diagnosis of ectopic breast tissue was made. Since the vast majority of ectopic breast tissue is found around the breast mound, axilla, and along the milk line, this case is peculiar in its location. This report shares our experience and provides indications for excision of incidental ectopic breast tissue.

## Introduction

Ectopic breast tissue (EBT) is an uncommon entity that occurs in about 0.2%–6% of the population (1, 2). EBT is an embryological abnormality that results from involutional failure of the mammary ridge, commonly referred to as the milk line (3). The milk line starts from the axilla and extends inferior to the groin, leading to the possibility of EBT along this axis. However, axillary and inframammary locations account for most EBT, and other sites are seldom reported. The histologic composition of EBT is also variable and needs further documentation. EBT harbors a wide spectrum of histological structures, from the complete formation of a breast unit, including the nipple-areolar complex, to only a rudimentary presence of pilosebaceous tissue (4). Most physicians recognize EBT as a small extra nipple (polythelia) or a lump in the axilla (polymastia), but the entire spectrum of EBT is much more variable and needs recognization.

Another concern with EBT is the possibility of malignant transformation. Primary breast cancer in EBT is very rare and is reported to represent only 0.3%–0.6% of all breast cancer. Due to their low incidence, when and how they turn into a malignant lesion and in which situations they need to be removed are not clearly defined (5). Because of the nonspecific characteristics of EBT, its low incidence, and the reluctance of patients to obtain a routine exam for EBT, data on EBT such as imaging, clinical course, and histology need more accumulation to investigate the correlation between other breast conditions further.

We present a 50-year-old female patient who presented initially with an inguinal bulge that mimicked an idiopathic benign neoplasm of the groin. However, excisional biopsy proved otherwise, with strong expression of breast-related immunohistochemical markers on pathology. In this case report, we share our experience of the workup, surgical treatment, and postoperative follow-up. A review of the latest literature for pertinent findings of this rare entity is also provided.

## Case Report

A 50-year-old woman visited our institution, complaining of rapid bulging on her left groin. The patient had recognized a small bump a year before but did not seek treatment. She denied any medical comorbidity, and she had no history of lymphoproliferative disease. Gynecological history was also nonspecific, other than a normal spontaneous vaginal delivery 25 years earlier. She was at her perimenopausal stage.

**Figure 1:**
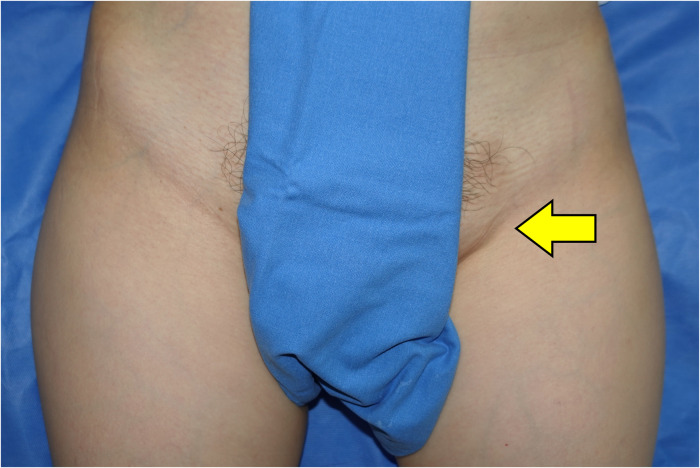
Preoperative findings: a 3 × 4-cm, soft, nontender, mobile, and palpated mass was found in the left inguinal area (arrow).

On physical examination, a soft, nontender, mobile, and palpable mass, 4 cm in size, was found in the inguinal area, 5 cm inferior to the left inguinal ligament (Figure 1). A Valsalva test to rule out inguinal hernia was negative. The acoustic Doppler exam revealed negative findings. A computed tomography (CT) scan was taken for further workup of this neoplasm.

**Figure 2:**
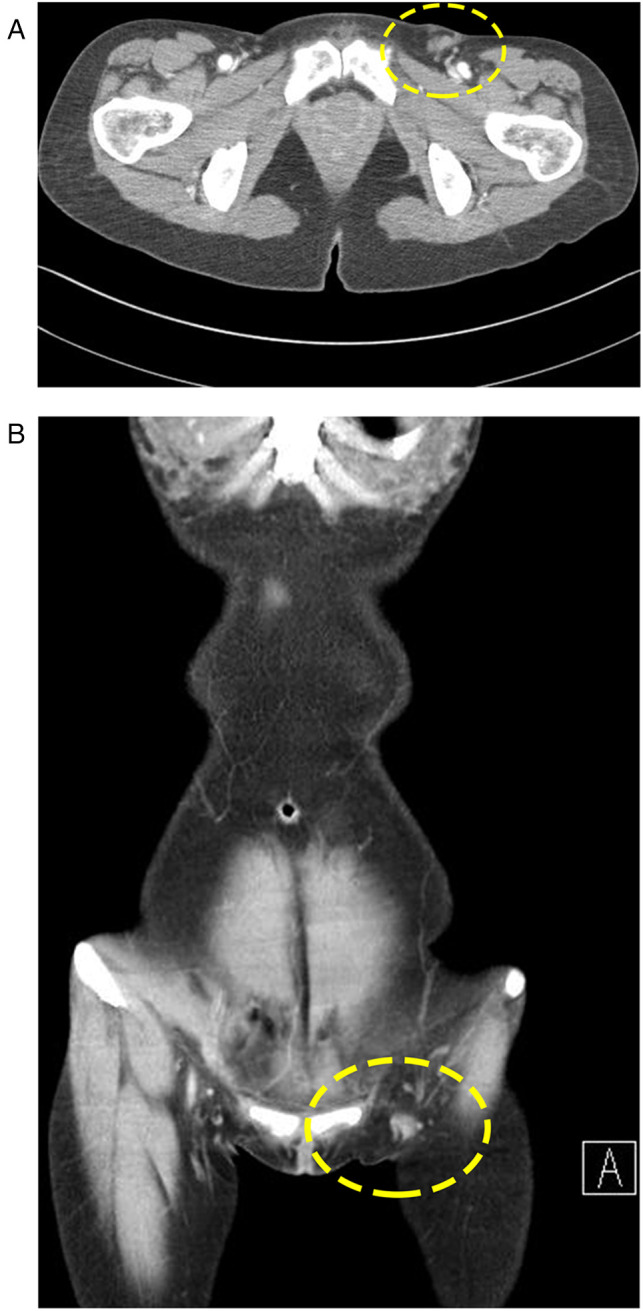
Preoperative CT scan: (**A**) A 1.7-cm, regional conglomeration of dense, soft tissue is evident. (**B**) Lesion in (A) is surrounded by benign-looking adipose tissue.

Abdominal CT showed an approximately 4-cm, heterogeneously enhancing, nodular neoplasm in the left inguinal area (Figure 2). Upon diagnosis of a benign soft tissue lesion such as lipoma, or a lymphoproliferative disease such as Kikuchi's disease, we planned an excisional biopsy of the mass.

**Figure 3:**
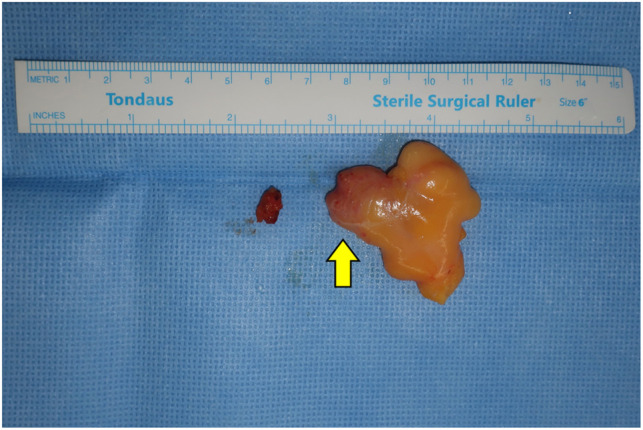
Gross finding: A single discrete rubbery mass with an enlarged lymph node: a solid portion is seen inside the fatty mass (arrow).

Excision was performed under local anesthesia. Upon dissection, the mass was readily visible under the scarpa's fascia layer. Blunt dissection was performed around the mass, and it was easily evacuated. Along with the main mass, a lymph node was identified and was also taken out for biopsy. On gross examination, the mass was about 5 cm in size, soft, and lipomatous in consistency (Figure 3). The specimen was sent for histologic examination.

**Figure 4:**
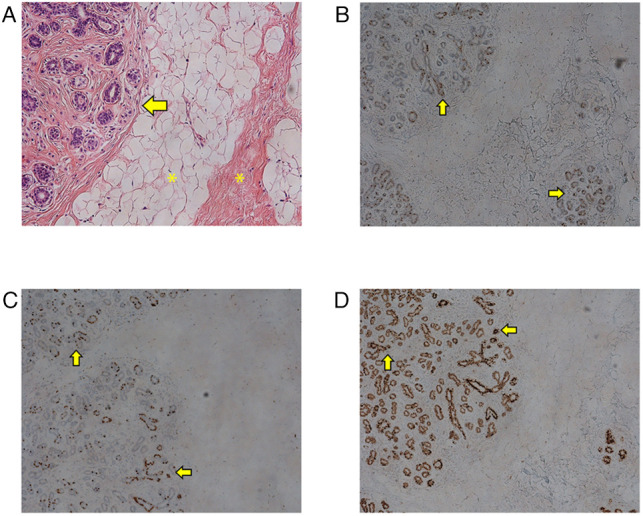
Microscopy findings: (**A**) Normal microscopic features of female breast tissue are evident. Multiple ducts lined by myoepithelial and epithelial cells (arrow) with surrounding stroma and adipose tissue (asterisks) are seen. (**B**) Immunohistochemistry (IHC) staining for estrogen receptor (ER) showing positivity throughout the whole glandular epithelium (arrow). (**C**) IHC staining for progesterone receptor (PR) showing positivity throughout the glandular epithelium. (**D**) IHC staining for GATA binding protein 3 showing positivity.

Histology showed a complete lobular structure surrounded by adipose and a collagenous stromal background, which is not a typical finding of a lipoma nor a lymphoproliferative lesion (Figure 4A). For further diagnosis, immunohistochemical (IHC) staining for apocrine and mammary markers was carried out. IHC for estrogen receptor (ER), progesterone receptor (PR), and GATA3 all showed positive expression (Figures 4B–D). The lymph node showed benign hyperplasia. Based on these findings, the diagnosis of EBT was made. Since the excision, the patient has been monitored without recurrence.

## Discussion

EBT is a well-known developmental anomaly that may develop along all sites of the mammary ridge (6). This entity is only partially recognized under the name of the supernumerary nipple or accessory breast, as EBT embodies a much wider spectrum (1). A classification suggested by Kajava thoroughly describes all clinical features of EBT with regard to the tissue composition and anatomical location. According to this classification, our case corresponds to a class IV EBT (Table 1).

**Table 1:** Kajava classification of ectopic breast tissue.

Class	Description
Class I (polymastia)	Complete breast(s) with the nipple, areola, and glandular tissue
Class II (supernumerary breast without areola)	Nipple and glandular tissue but no areola
Class III (supernumerary breast without nipple)	Areola and glandular tissue but no nipple
Class IV (mamma aberrata)	Glandular tissue only
Class V (pseudomamma)	Nipple and areola but without glandular tissue (replaced by fat)
Class VI (polythelia)	Nipple only
Class VII (polythelia areolaris)	Areola only
Class VIII (polythelia pilosis)	Patch of hair only

EBT in the inguinal region has been reported in previous literature (7, 8). However, the diagnostic course is a noteworthy feature of our experience. On clinical examination, only a soft, nontender lump was seen. Due to its low incidence, we could not associate our clinical findings with a rare entity such as EBT. Class I–III EBT can be easily diagnosed with clinical exams due to the presence of nipple or areolar structures (9). Most axillary EBTs are diagnosed with ultrasound, but this is possible only when there is a high degree of suspicion for EBT. A peculiar location such as the inguinal area greatly obscures the impression of EBT. In cases of atypical locations outside the milk line, as in this case, a modified development of sweat glands, rather than the migratory arrest of breast primordium, can also be suggested as the origin of the lesion (10).

Most reports describe sonographic findings of EBT, and reports of CT or MRI imaging of EBT are scarce (7, 11). In our case, the peculiar location of the lump led us to perform a CT scan. Our CT scan did not reveal a sizable glandular portion. It revealed only a nonspecific cluster of lymph nodes and fat accumulation in the corresponding area, which were not suggestive of EBT. In this case, histology was the only and last cue for the right diagnosis. H&E staining showed an unexpected glandular structure, complete in its form, among the adipocollagenous stroma. IHC staining showed positivity for ER, PR, and GATA3. Considering the atypical location of the breast tissue, IHC for GATA3 was important to rule out the possibility of metastatic carcinoma. Since GATA3 is normally expressed in apocrine structures, the existence of GATA3-positive EBTs might support the statement that EBT can arise *de novo* from an apocrine structure at its native location, rather than through the conventionally hypothesized migratory arrest (10).

The proper indications for surgical removal of EBT are unclear. Due to the rarity of EBT, the literature consists mostly of anecdotal case reports post excision and thus does not provide substantial evidence or detailed analysis to suggest mandatory removal. Rationales for removal of EBT are mostly concentrated on the hypothetically higher risk of malignancy due to late presentation, with less chance of evaluation (7, 12). Also, early removal is advantageous and less invasive since it is prophylactic (13). A notable study by Fama et al., in which they analyzed their experience with 327 EBTs, concluded that this risk of malignancy arising from EBT is not negligible and that they should be recognized as candidates for surgical removal, especially the Kajava class I–IV lesions (2).

In line with the prior literature, the authors would like to suggest judicious excision in cases such as ours that exhibit clinical features such as (1) a middle-aged woman, particularly in her fifth or sixth decade, when the incidence of breast cancer peaks; (2) sustained growth unrelated to the menstrual cycle; (3) Kajava class I–IV; and (4) an indistinct impression on physical examination and even with imaging workup. The possibility of malignancy should always be explained to the patient.

This study has its limitations. We report an anecdotal experience of a very rare occurrence that cannot be generalized to all cases of the same entity. We have not been able to review all the literature.

## Conclusion

EBT is rare and is usually found along the so-called “milk line,” which is an embryological trail of breast buds along the anterior axilla and trunk. The vast majority of EBT is found in the upper chest and axilla. Other sites are seldom reported, and further reports are needed. In typical locations such as the axilla, EBT diagnosis is not difficult and is usually followed up with ultrasonography. In the present case, however, the EBT was located in the inguinal canal, making the excisional biopsy a crucial step in confirming the diagnosis.

Due to the paucity of previous research, prophylactic excision of all EBTs cannot be fully supported. However, the fact that EBT in occult locations is hardly detectable without a pathologic symptom is a critical reason to support its surgical excision. Therefore, the option of prophylactic excision of EBTs can be given considerable weight by the surgeon in select cases when a comprehensive assessment of patient demographics and anatomical location is supportive. In this situation, the risk of malignant change will be safely eliminated.

## Data Availability

The original contributions presented in the study are included in the article/Supplementary Material, further inquiries can be directed to the corresponding author.
